# Contribution of a novel gene to lysergic acid amide synthesis in *Metarhizium brunneum*

**DOI:** 10.1186/s13104-022-06068-2

**Published:** 2022-05-18

**Authors:** Kelcie N. Britton, Chey R. Steen, Kyle A. Davis, Jessi K. Sampson, Daniel G. Panaccione

**Affiliations:** Division of Plant and Soil Sciences, West Virginia University, Morgantown, WV 26506 USA

**Keywords:** Ergot alkaloids, α/β hydrolase fold protein, Lysergic acid amides, *Metarhizium*

## Abstract

**Objective:**

The fungus *Metarhizium brunneum* produces ergot alkaloids of the lysergic acid amide class, most abundantly lysergic acid α-hydroxyethylamide (LAH). Genes for making ergot alkaloids are clustered in the genomes of producers. Gene clusters of LAH-producing fungi contain an α/β hydrolase fold protein-encoding gene named *easP* whose presence correlates with LAH production but whose contribution to LAH synthesis in unknown. We tested whether EasP contributes to LAH accumulation through gene knockout studies.

**Results:**

We knocked out *easP* in *M. brunneum* via a CRISPR/Cas9-based approach, and accumulation of LAH was reduced to less than half the amount observed in the wild type. Because LAH accumulation was reduced and not eliminated, we identified and mutated the only close homolog of *easP* in the *M. brunneum* genome, a gene we named *estA*. An *easP*/*estA* double mutant did not differ from the *easP* mutant in lysergic acid amide accumulation, indicating *estA* had no role in the pathway. We conclude EasP contributes to LAH accumulation but is not absolutely required. Either a gene encoding redundant function and lacking sequence identity with *easP* resides outside the ergot alkaloid synthesis gene cluster, or EasP plays an accessory role in the synthesis of LAH.

**Supplementary Information:**

The online version contains supplementary material available at 10.1186/s13104-022-06068-2.

## Introduction

Ergot alkaloids derived from lysergic acid have impacted humankind for millennia as toxins in contaminated grain crops and as pharmaceuticals to treat dementia, migraines, hyperprolactinemia, and other disorders [1–3]. Many pharmaceutical ergot alkaloids are derivatives of lysergic acid amides. Naturally occurring lysergic acid amides include ergonovine (called ergometrine in Europe) and lysergic acid α-hydroxyethylamide (LAH). Ergonovine is the primary lysergic acid amide in the rye ergot fungus *Claviceps purpurea*, which also produces abundant ergopeptines [2, 4]; whereas, the plant root symbiont and insect pathogen *Metarhizium brunneum* produces ergonovine and LAH, with the concentration of LAH dwarfing that of ergonovine by approximately 200 to 1 [5, 6]. In studies to date, all genes required for ergot alkaloid synthesis are clustered in the genomes of the producing fungi in ergot alkaloid synthesis (*eas*) clusters [7–12].

**Fig. 1:**
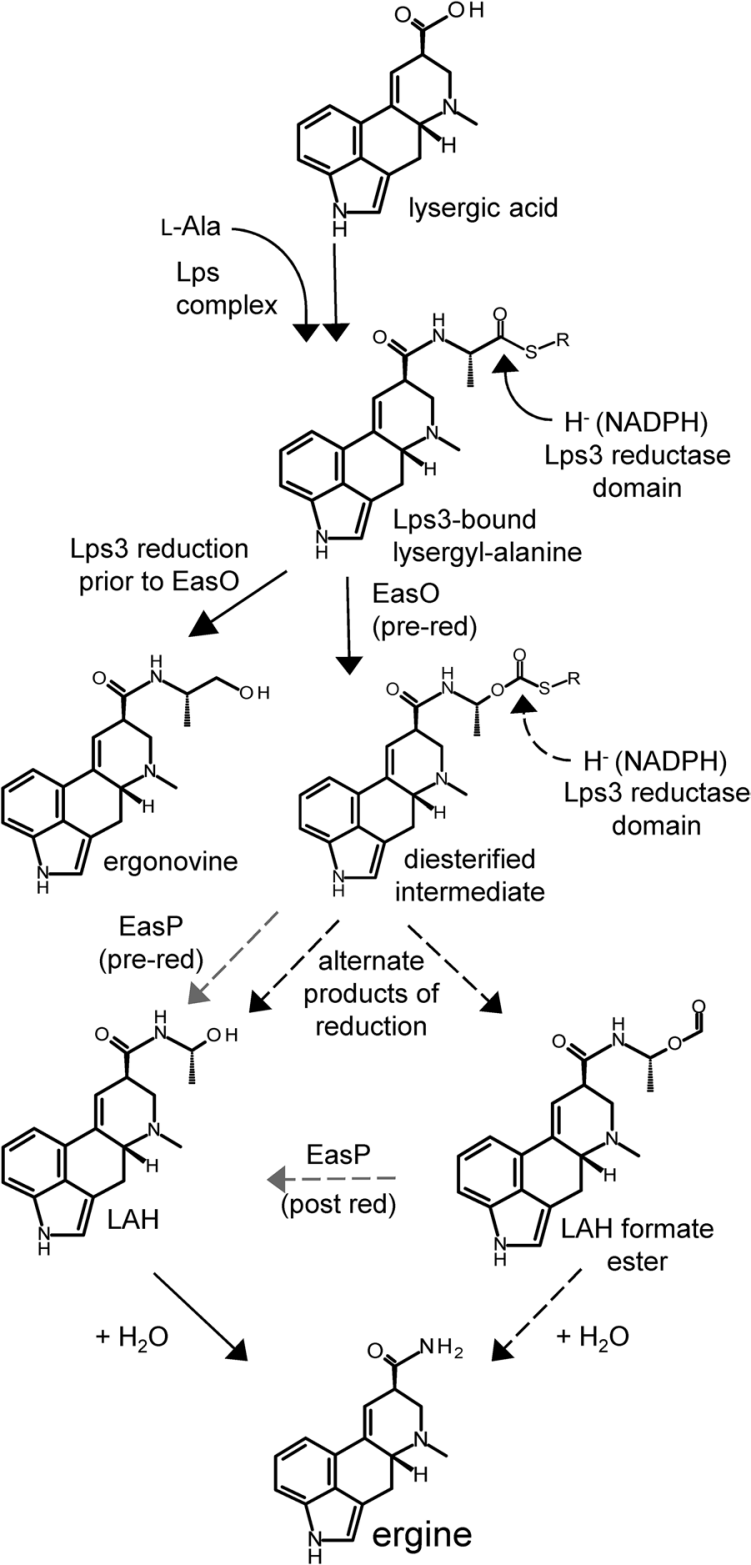
Pathway to lysergic acid amides. Enzymes demonstrated (or hypothesized, in the case of EasP) to have roles are indicated at relevant steps. LAH, lysergic acid α-hydroxyethylamide; red, reduction by reductase domain of Lps3; R, to Lps3 via pantetheine. Dashed arrows indicate hypothesized steps, with those involving EasP shown in gray

Both ergonovine and LAH are derived from the intermediate lysergyl-alanine [4, 6] which is synthesized via a complex of two monomodular peptide synthetases, lysergyl peptide synthetase (Lps) 2 and Lps3. Lps2 recognizes lysergic acid, activates it by adenylation, binds it as a thioester, and condenses it with alanine that has been similarly recognized, activated, and thioesterified by Lps3. Lps3 contains a carboxy terminal reductase domain that can reduce thioesterified lysergyl-alanine to ergonovine [4]. The reductase domain uses hydride ions obtained from its cofactor NADPH to reduce the carbonyl carbon of the alanyl moiety of lysergyl-alanine to an aldehyde and then a primary alcohol, liberating ergonovine from enzyme bound lysergyl-alanine [4] (Fig. 1).

In fungi that produce LAH as well as ergonovine (e.g., *M. brunneum*, the paspalum ergot fungus *Claviceps paspali*, and the morning glory symbiont *Periglandula ipomoeae*) there are two additional genes in the *eas* cluster: *easO*, encoding a Baeyer–Villiger monooxygenase (BVMO) required for synthesis of LAH [6], and *easP*, encoding an α/β hydrolase fold protein, a role for which in ergot alkaloid synthesis has not yet been demonstrated but whose presence correlates perfectly with the ability to produce LAH [7, 8, 10]. As a BVMO, EasO is hypothesized to insert an oxygen between the alpha carbon and carbonyl carbon of the alanyl portion of lysergyl-alanine [6]. Gene knockout and stable-isotope labeling data support this hypothesis. The hypothesized intermediate produced by EasO provides a substrate that could be converted to LAH through the activity of a carboxyl esterase (potentially an α/β hydrolase fold protein encoded by *easP*) or through the activity of the reductase domain of Lps3 (Fig. 1) [6]. In the present study, we mutated *easP* through a CRISPR/Cas9-based approach to test its contribution to synthesis of lysergic acid amides.

## Main text

### Materials and methods

*Metarhizium brunneum* ARSEF 9354 was cultured at 30° C on sucrose yeast extract agar or malt extract broth [5]. To maximize ergot alkaloid yield, 20 µL of a conidial suspension (40,000 conidia per µL) were injected into larvae of the model insect *Galleria mellonella* [5, 13].

The *easP* locus of *M. brunneum* was knocked out via a transient CRISPR/Cas9-mediated approach based on the protocol described by Davis et al. [14]. An sgRNA was synthesized from the template 5′-TTCTAATACGACTCACTATAGTCTGCTCCATGGAGGCTCCTGTTTTAGAGCTAGA-3′ (the 20-nt target sequence is underlined and an additional G was inserted immediately preceding the target sequence) with the EnGen sgRNA synthesis kit (New England Biolabs, Ipswich, MA, USA). The sgRNA was complexed with EnGen Spy Cas9 NLS (New England Biolabs) and co-transformed into protoplasts along with a phosphinothricin-resistance conferring fragment [14]. Transformants were screened for mutations at *easP* in PCRs primed with oligonucleotides PcrspF (5′-CACACTCTACTCCCTCACAAGG-3′) and PcrspR (5′-CCGCTCCAGGCATCGTCAC-3′). Reaction conditions included an initial denaturation at 98 °C for 30 s, followed by 35 cycles of 98 °C for 15 s, 66 °C for 15 s, and 72 °C for 80 s. PCR products that differed in length from that of wild-type *M. brunneum* were Sanger sequenced at Eurofins Genomics (Louisville, KY, USA).

The *estA* gene was knocked out via a similar approach, except the template for the sgRNA was 5′-TTCTAATACGACTCACTATAGTCCAAGACGTAGCCGACTTCGTTTTAGAGCTAGA-3′ (where the target sequence is underlined and preceded by an additional G), and the selectable marker was the hygromycin resistance-conferring plasmid pBC-hygro [15]. Transformants were screened by PCR with oligonucleotides *estA*F (5′-GAACACAACTTCACCACATCGC-3′) and *estA*R (5′-GACGTCACCGAGCTCTCTG-3′) in reactions with conditions as described above, except the annealing temperature was 64 °C and the extension time 180 s.

Ergot alkaloids and ergosterol were extracted from infected larvae of *G. mellonella* by bead beating in methanol [5, 6]. To detect ergot alkaloids, 20 µL of extract was analyzed by high performance liquid chromatography (HPLC) with fluorescence detection (excitation 310 nm/emission 410 nm) [5, 6, 16]. Ergot alkaloid quantities in each sample were normalized to the quantity of the fungus-unique sterol ergosterol in that same extract as described by Steen et al. [6]. Data were compared by Brown-Forsythe tests to assess equality of variances. Data that passed a Brown-Forsythe test (*P* > 0.05) were analyzed by single-factor ANOVA. For data sets involving multiple comparisons, a Tukey–Kramer Honestly Significant Difference test was employed. In three comparisons (ergonovine and lysergyl-alanine in the *easP* knockout compared to wild type, and ergine in the *easP*/*estA* double knockout), data did not pass a Brown-Forsythe test (*P* < 0.05) and subsequently were compared nonparametrically, initially with a Wilcoxon rank sums test and then (for the ergine data) a Steel–Dwass nonparametric multiple comparison test. Statistical analyses were conducted in JMP Pro 14 (SAS, Cary, NC, USA).

### Results

**Fig. 2:**
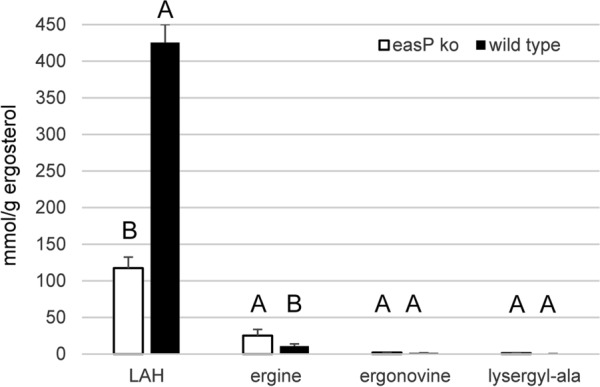
Accumulation of LAH and other lysergic acid amides in wild-type *M. brunneum* versus an *easP* knockout strain. Extracts were prepared from larvae of the model insect *Galleria mellonella* seven days after infection with the indicated strains. Moles of ergot alkaloids were measured relative to a standard curve of ergonovine and normalized to mass of the fungus-unique sterol ergosterol as a surrogate for fungal biomass. Bars indicate means (n = 6 fungus-infected larvae), and error bars indicate standard error. Different letters within an alkaloid comparison indicate that fungal strain had a significant effect on accumulation of the indicated lysergic acid amide (*P* < 0.05)

Introduction of Cas9 complexed with an sgRNA targeting *easP*, which was co-transformed with a selectable marker for phosphinothricin resistance, resulted in knockout of *easP*. As a haploid fungus, *M. brunneum* contains only a single allele of *easP*. Sequence analysis of the *easP* locus in two independent transformants indicated Cas9 cut three bp before the PAM site and a portion of the cotransformed phosphinothricin-resistance marker was ligated into the *easP* locus during repair (Additional file 1: Figure S1). Knockout of *easP* had a significant effect on accumulation of LAH relative to fungal biomass (estimated by measuring the fungus-unique sterol ergosterol) in *M. brunneum*-infected larvae of the model insect *G. mellonella* (Fig. 2). The *easP* knockout resulted in a significant decrease (approximately 70%) in accumulation of LAH (*P* < 0.0001). The concentrations of alternate lysergic acid amides, ergonovine and lysergyl-alanine were not affected in the *easP* knockout. Ergine, the simple amide of lysergic acid, accumulated to a higher concentration in the *easP* knockout than in the wild type (*P* = 0.003). Ergine can arise as a spontaneous hydrolysis product of LAH [17, 18] (Fig. 1) and also from hydrolysis of other lysergic acid derivatives [19]. A similar biochemical phenotype was observed qualitatively in the second *easP* knockout. Whereas the primary purpose of this study was to assess the contribution of *easP* to LAH biosynthesis, the *easP* knockout also was investigated for changes in radial growth and sporulation in vitro and colonization of *G. mellonella* larvae (measured by ergosterol accumulation). No significant differences were detected in these traits (Additional file 2: Table S1).

**Fig. 3:**
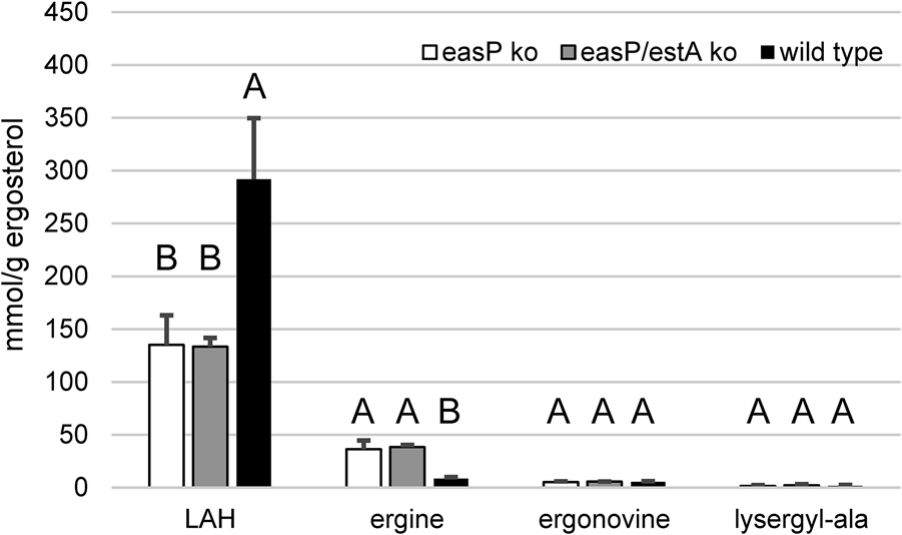
Effect of *estA*/*easP* double mutation relative to *easP* mutation on accumulation of LAH and other lysergic acid amides in *M. brunneum*. Alkaloids are expressed as moles of ergot alkaloids per mass of the fungus-unique sterol ergosterol as a surrogate for fungal biomass. Data represent means (n = 6 fungus-infected *G. mellonella* larvae), and error bars represent standard error. Extracts were prepared seven days after infection. Strains, within an alkaloid type, marked with different letters differed significantly (*P* < 0.05)

Because the *easP* knockout mutation led to reduction as opposed to elimination of LAH, we investigated the possibility that a similar enzyme with redundant function was encoded in the *M. brunneum* genome. Since the *eas* cluster contained no additional genes with unidentified function, we queried the haploid genome of *M. brunneum* ARSEF 3297 for genes with the capacity to encode proteins similar to EasP by tblastn search. The *M. brunneum* genome encoded only one additional significant match (E value 2e^−05^; no other match had an E value less than 1). The homolog’s translation product corresponds to the α/β hydrolase fold protein under GenBank accession XP_014542068. We named this gene *estA*, for esterase A, and knocked it out by a CRISPR/Cas9 approach in the *easP* knockout strain while selecting for a co-transformed hygromycin resistance marker (Additional file 3: Figure S2). Knockout of *estA* did not affect ergot alkaloid accumulation relative to the e*asP* knockout (Fig. 3), indicating that EstA plays no role in the ergot alkaloid pathway. In this data set, LAH was reduced by a mean of 54% in the *easP* and *easP*/*estA* knockouts relative to wild type. Concentrations of ergonovine and lysergyl-alanine did not differ significantly among strains. The concentration of ergine was again higher in the *easP* knockout (and in the *easP*/*estA* double knockout) than in the wild type, with *P* values of 0.035 and 0.014, respectively, for the two *easP* mutant strains compared to the wild type in a Steel–Dwass multiple comparison test.

## Discussion

Our data indicate that EasP contributes significantly to LAH accumulation but is not absolutely required. One hypothesis to account for this observation is that a similar enzyme encoded in the *M. brunneum* genome has a redundant function. Functional analysis of the only homolog of *easP* in the *M. brunneum* genome did not support this hypothesis. If another enzyme is performing a function redundant with that of EasP, the enzyme must be unrelated in terms of amino acid sequence and also encoded outside the *eas* cluster. To date all genes required for ergot alkaloid synthesis have been encoded in the *eas* cluster [2, 3, 7, 8]. In a recent study relevant to this point, Jones et al. [10] reported that three species of *Aspergillus* synthesize LAH and evolved the steps for incorporation of lysergic acid into LAH independently of *M. brunneum* (and other fungi in the Clavicipitaceae) yet had similarly constituted *eas* clusters with no additional genes present that might be redundant with *easP*. The possibility that two lineages evolved similar pathways for incorporating lysergic acid into LAH yet kept one unrelated and redundant gene outside the *eas* cluster appears unlikely.

An alternate hypothesis to account for the remaining LAH in the *easP* knockout backgrounds is that EasP plays an accessory role in generating LAH and that LAH can be synthesized in the absence of EasP or any carboxyl esterase. Steen et al. [6] suggested the reductase domain of Lps3 might liberate LAH from a carboxyl ester/thioester diester intermediate arising from the activity of the Baeyer–Villiger monooxygenase EasO on Lps3-bound lysergyl-alanine (Fig. 1). Liberation of LAH via reduction of the carbonyl carbon of this intermediate could produce LAH without the requirement for esterase activity. The reductase domain of Lps3 reduces that same carbonyl carbon during the production of ergonovine in *C. purpurea* [4]. Although not required for LAH synthesis in this scenario, EasP might still contribute to LAH accumulation via one or more of several possible mechanisms. One mechanism would be for EasP to hydrolyze LAH from Lps3-bound carboxyl ester/thioester derivative of lysergyl-alanine (Fig. 1) prior to reduction by the Lps3 reductase domain. A second mechanism by which EasP could increase LAH accumulation via this same model would be to salvage LAH from an LAH formate ester that would result if the activity of the reductase domain of Lps3, acting on that same carbonyl carbon, resulted in bond breakage on the thioester side of the diester intermediate. The resulting formate ester might be hydrolyzed to LAH by EasP acting as a carboxyl esterase. No novel fluorescent peaks stood out in the *easP* knockout extracts, but increased ergine noted in *easP* mutants may have arisen at least in part from hydrolysis of lysergic acid amides other than LAH which, as a typical source of ergine, was present in lower concentration in the *easP* knockout backgrounds. Testing these hypothetical roles for EasP in conjunction with Lps3 will require mutation of the reductase domain of Lps3 while retaining the earlier functions of the enzyme (adenylation and thiolation of alanine). Such experiments are the objectives of future work in our laboratory.

## Limitations

We showed EasP contributes significantly to LAH accumulation, but we have not demonstrated the mechanism by which it acts or identified the origin of the remaining LAH in the *easP* knockout. We lack the genetic resources to test whether the remaining LAH in an EasP mutant results from activity of the reductase domain of Lps3. Investigation of whether a gene outside the *eas* cluster is required for synthesis of the remaining LAH will require novel approaches. Our study focused on the contribution of *easP* to LAH biosynthesis; we did not investigate its potential contribution to other pathways.

## Supplementary Information

**Additional file 1: Figure S1.** Disruption of the *easP* locus of *M. brunneum*.

**Additional file 2: Table S1.** Effect of *easP* mutation on radial growth, sporulation, and insect colonization.

**Additional file 3: Figure S2.** Disruption of *estA* in the *easP* knockout of *M. brunneum*.

**Additional file 4.** Spreadsheet containing data analyzed in Figs. 2 and 3 and Additional file 2: Table S1.

## Data Availability

All data generated or analyzed during this study are included in this published article and its additional files (Additional files 1 through 4).

## Abbreviations

ANOVA: Analysis of variance
ARSEF: Agricultural Research Service collection of entomopathogenic fungi
BVMO: Baeyer–Villiger monooxygenase
Cas9: Clustered regularly interspaced palindromic repeats-associated protein 9
CRISPR: Clustered regularly interspaced palindromic repeats
Eas: Ergot alkaloid synthesis
HPLC: High performance liquid chromatography
LAH: Lysergic acid α-hydroxyethylamide
Lps: Lysergyl peptide synthetase
NLS: Nuclear localization sequence
PCR: Polymerase chain reaction
sgRNA: Single guide RNA
