# Supplementary material for: Contribution of a novel gene to lysergic acid amide synthesis in Metarhizium brunneum
Source: BMC Res Notes. 2022 May 18;15:183. doi: 10.1186/s13104-022-06068-2 (PMC9118626; doi:10.1186/s13104-022-06068-2)
Supplement: Supplementary file 1 — Additional file 1: Figure S1. Disruption of the easP locus of M. brunneum. [file 13104_2022_6068_MOESM1_ESM.pdf]

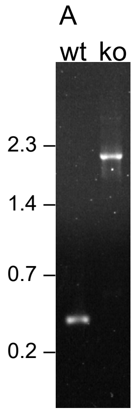

B

GCCGAGGCTTTCCATGCGTGGCTGCACTCCTCATTCCCCAAGGTACTGGCCGAGAGG
GCGCCAGGCTTCCAGGTCGATTTGTCGCGGATTCTGCTCCATGGAGGCTTGGAGTAT
GTCTGCACGTTTTGAGTGATTTATCATGGCTATTACTCGCGGTCTTGCTGCCAGCAG
GCATATCTACAACACGATAAGGCCAGCAGGGCCTACCACGGGGGTACATGGACGCCA
GGCTGCGACAACCATGGAACAGCGGGTACCCCTCATGTCCATGTTTTTTTTCTCTCT
CAAGGTGGTGCTCATGTGCTCGTCGGTGTGGTTGAGAGAGCGTTTCTATTAACGAAT
TGTGTCCCCGCCGGATCCGTAAANNNNNTTTTCGAACCTGGCCAAAACTGAAGTGTC
TATTTCTTCATGTAGTCATGCTGGCTGCAGGACCTTACCAGGGAGGAAGTTTCCGGT
CTTCGAACGAATGTTGGGAAACTGAAAACTGGAAAAATTAGTATAGAAAGAAAGTTG
CTGTTGCGATATCTTGATATATCAGTGCACCCTGACAAAGCAGCATCCGTATCTCAC
CAATCTACCAGAAGCATACCTCGATGGTCTCAACTTAGCGGCGAAATTTGCTATGGA
ATGTATGTCAGATTCTGCCTCCT**CGG**GTGCTTACCTCGCCCTGTCTTCTGCTCTGGC
GCACCCAGACTCTTACCGGTCCCTCAGTCTTATGTATCCGATGATCGATTTCGACAC
CGACTGGTGGCGCCGAGGCAGCCGAGCTGTCGGTGCGCCAAACCCAGGCTGCTTGCC
GGATGCCGCGTTCCCTGCAAATGACAATACTGTCAAGGACCAGC

Fig. S1. Disruption of the *easP* locus in *M. brunneum*. (A) PCR products from genomic DNA of *M. brunneum* ARSEF 9354 (wt) and an *easP* knockout (ko) primed with oligonucleotides PcrspF and PcrspR (section 2.2) flanking the site of Cas9-initiated recombination. Relative mobility of relevant fragments (lengths in kb) of *Bst*EII-digested bacteriophage λ are shown at left. (B) DNA sequences flanking the site of recombination of *easP* locus after CRISPR-Cas9 mutagenesis. Sequences from the DNA fragment conferring phosphinothricin resistance, which were incorporated into the locus during repair, are shaded gray. The target sequence of the sgRNA is underlined, and the PAM site is shown in bold. The characters NNNN represent 1000 nt omitted to simplify the presentation.
