# Supplementary material for: Contribution of a novel gene to lysergic acid amide synthesis in Metarhizium brunneum
Source: BMC Res Notes. 2022 May 18;15:183. doi: 10.1186/s13104-022-06068-2 (PMC9118626; doi:10.1186/s13104-022-06068-2)
Supplement: Supplementary file 2 — Additional file 2: Table S1. Effect of easP mutation on radial growth, sporulation, and insect colonization. [file 13104_2022_6068_MOESM2_ESM.pdf]

Table S1. Effect of *easP* mutation on radial growth, sporulation, and insect colonization[a]

Metarhizium brunneum strain	Colony diameter (mm), day 6 (n=6)	Conidia/mm^2 culture, day 6 (n=6)	Insect colonization (µg ergosterol/larva), day 7 (n=14)
wild type	25.8 ± 0.3 A[b]	1306 ± 62 A	96.1 ± 7.8 A
easP knockout	26.0 ± 0.4 A	1310 ± 101 A	85.0 ± 8.3 A

[a]data represent mean ± standard error

[b]values followed by the same letter within a column do not differ significantly in ANOVA; *P* values 0.76 for colony diameter, 0.97 for conidiation, and 0.82 for insect colonization
