# Supplementary material for: Contribution of a novel gene to lysergic acid amide synthesis in Metarhizium brunneum
Source: BMC Res Notes. 2022 May 18;15:183. doi: 10.1186/s13104-022-06068-2 (PMC9118626; doi:10.1186/s13104-022-06068-2)
Supplement: Supplementary file 3 — Additional file 3: Figure S2. Disruption of estA in the easP knockout of M. brunneum. [file 13104_2022_6068_MOESM3_ESM.pdf]

A

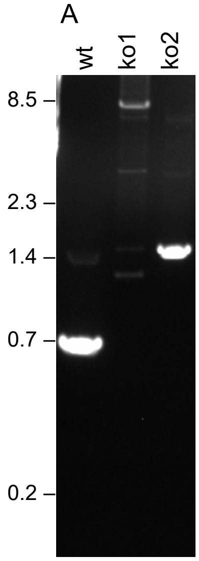

B

ATGGCCTCCAAACTCCAGGGCCTCAAGGTCCTCCAAACGCCCTACAAAACGATTGCCCACCACGAAATCC
GCACCGACGTCCTCGTCCCCGAAACGCCACTGTCCGGCAAACGACCCGTCATCGGCGGCGCCTACAATCC
ATGCCAACCCGTTCCATGTGCTCGCCGAGGCGGCATAAATCGCCGTGACGATCAGCGGTCCAATGATCGA
AGTTAGGCTGGTAAGAGCCGCGAGCGATCCTTGAAGCTGTCCCTGATGGTCGTCATCTACCTGCCTGGAC
AGCATGGCCTGCAACGCGGGCATCCCGATGCCGCCGGAAGCGAGAAGAATCATAATGGGGAAGGCCATCC
AGCCTCGCGTCGAGCTTTGAAGTTGCTGCAAGCTGGCTTCAAGCCATCCCATCCGAATGTGATGGATGCG
TTCTTTCTGGGCCGTTGCGACTTTGGGGATCGTCTTTCCCGCGCCCTTGGTTGGAGGCCCTGTCTCCGGT
GTCCCTTGTCCCTTCCAGGCAAGCGAGCGAGGTCCATTCAGATGGTGCTCCATCAGCGTTGGCTTTCCGT
CTCCATTGGCTCTTGGCAATTCGGTCAGCGGGGCTGACTGCCTCAGGTGGGGCAGTGCTAGTGTGTGTAC
CGACCCGCAGGATTGGTGCTTTGCCCAGAGCTCTACAGAATAGCGCGCGCATCCATATGTTAGTTCTGCA
ATTTTCTTGTATCGGTGCTGTGACTCATACTTCCCCCTTTGGCTGGCCTTGCGGCAACCAATAAGAACGC
ACAGTGAAATCTTGCGGGTGGGGAGTGGATCCATGGCGCCTGCATTGGCTTGGGGACGCGCACTGTCGCA
CACTTCCATCTGACCTTTCAGAAGGGTTTCGTGGTGGGCAAGGACCAACCGGTTGCGCGGCCGTGCGTGG
GTGCCTCGCCCGGCACTGCCAGGGCCACTGCAGTGGCAGTTTGCTGCCTGATACAAAATCCTTCCCTCCG
CCCAGTTTTCCCTCTTTGACCTTCCTTTCTCTTCTCTGCAACCAAATCCACCCTATCAAACCAAAACAGT
ATCTCGACCGAGGTATCAACCTGAATCAGCAACATCGTAGCCAGCATTTGTCTCCGTCTCTGCAGAACCA
GCGAGTTGCAAACATTATCTTC**TGG**GCCTACATCCACTCGCCCTCGCTCGCCTCTCTCCTCTCCTCGCAG
TCGCCCCCCGTCGAGGCCGACCTCGCCCGCGTCCTCGTGACCGGCGACTCGGCCGGCGGCCTCCTCTGCC
TAAGCACCGCGCTCACGTACCCGCGCGACGTGAGGGTCGCCGTCGCCACGTACCCCCTCGTCGACCCGGG
CGCGCCAGAGTTCACAACGCCCCGTGAGAACCCGCCGCTGGGGAAGAGCGTCCCGGAGTCGGTGTACGAC
GACGCGCTTGCCGCGGCGGAGGGCGGGCCGGCAGAGAGCTCGGTGACGTCCGCTGCGAGATT

Fig. S2. Disruption of *estA* in the *easP* knockout of *M. brunneum*. (A) PCR products from *M. brunneum* ARSEF 9354 (wt) and two *estA* knockouts (ko); *estA* knockout 2 was used in all subsequent studies. Template was genomic DNA from the indicated strains, and primers were oligonucleotides *estA*F and *estA*R (refer to section 2.2) flanking the site of Cas9-initiated recombination. Lengths of relevant fragments (in kb) of *Bst*EII-digested bacteriophage λ are indicated to the left of the gel. (B) Sequence of the *estA* knockout 2 locus after mutagenesis; sequences of the hygromycin-resistance conferring fragment (inserted during repair) are shaded gray. The remaining three nucleotides of the sgRNA target sequence is underlined, and the PAM site is bold; a deletion of 231 bp eliminated sequences corresponding to the remainder of the target sequences in the sgRNA.
